# The behaviour of *Escherichia coli* and *Pseudomonas aeruginosa* in bottled mineral water

**DOI:** 10.1016/j.heliyon.2023.e21634

**Published:** 2023-10-30

**Authors:** Michael Schalli, Sabine Platzer, Doris Haas, Franz F. Reinthaler

**Affiliations:** a Department for Water-Hygiene and Micro-Ecology, D&R Institute of Hygiene, Microbiology and Environmental Medicine, Medical University of Graz, 8010, Graz, Austria; b Applied Hygiene and Aerobiology, D&R Institute of Hygiene, Microbiology and Environmental Medicine, Medical University of Graz, 8010, Graz, Austria

**Keywords:** *Escherichia coli*, *Pseudomonas aeruginosa*, Carbon dioxide, Mineral water, Water supply, Food contamination

## Abstract

Microbial contamination of bottled water during the filling and capping procedure is a problem which should be avoided. The examination of the influence of carbon dioxide (CO_2_) on bacterial growth of *Escherichia coli* (*E. coli*) and *Pseudomonas aeruginosa* (*P. aeruginosa*) in bottled mineral water was the aim of this study. Commercially available glass bottles with plastic screw caps filled with natural mineral water (without additional CO_2_ “still” (StMW) and with CO_2_ “sparkling” (SpMW) were obtained from a manufacturer in the province of Styria, Austria. The artificial contamination was performed in the lab by opening the bottle with subsequent addition of a bacterial solution with a defined number of bacteria. For each bacterial strain, 12 bottles were prepared. Samples (100 mL) were taken after a specific number of days, filtrated and placed on Endo Agar for cultivation. After incubation for 24 h bacterial colonies were counted. In this study CO_2_ addition to bottled water reduced colony forming units of the two investigated bacterial strains over time.

## 1 Introduction

Bottled drinking water is not completely free of microorganisms. The autochthonous flora of the water source is of little concern to healthy people, but the process of processing and filling can cause intake of different pathogenic bacteria, viruses and fungi [[1], [2], [3]]. *Pseudomonas aeruginosa* (*P. aeruginosa*), a rod-shaped Gram-negative facultative aerobe bacterium, prefers to use oxygen as the final electron acceptor. It is also capable of using alternative electron acceptors such as nitrate [4]. Various acute and chronic water associated infections are caused by *P. aeruginosa*, which makes this bacterium a versatile target for investigations [5]. The opportunistic pathogen *Escherichia coli* (*E. coli*) is the predominant aerobic bacterium of the gut microbiota [6] and is present as an indicator-bacterium in faecal-contaminated water resources [7].

Providing safe drinking water is one of the most important hallmarks of a successful society. With contaminated drinking water the full potential of a community cannot be realized and public health is at risk [8]. A recent published study indicated, that over half (63.9 %) of Australian participants included in the study were consuming bottled water in the past week. Regular consumption was prevalent for fruit juices (38.8 %) followed by bottled water (37.4 %) [9]. The consumption of carbonated beverages led to an increased food ingestion with a heightened risk of weight gain, obesity and fatty liver disease in mice. In their study, Eweis et al. proposed, that the intake of carbonated beverages led to an accumulation of CO_2_ in the stomach, which results in a signal to the brain that stimulates the hunger sensation [10]. The intake of carbonated drinks can have positive effects on the digestive process with a slight increase of hydrochloric acid, or can worsen an acid related disease [11]. Extended bottled water consumption with limited resources is connected with high amounts of packing material needed for storage. An extensive replacement of glass materials with plastic for food packaging for beverages and bottled water occurred in the last decade [12]. Microbial contamination during the process of consumption of beverages can lead to extended bacterial and fungal growth in unfinished bottles. Ingredients like carbon dioxide (CO_2_) and organic matter, were found to be important factors for microbial growth in beverages [13]. An easy method for the detection of bacterial growth in water is heterotrophic plate count (HPC), which was performed to enumerate bacterial counts [14,15]. Duranceau et al. (2012) examined the influence of storing conditions of bottled water on HPC. For bottled water stored inside the house (24 °C) or in a fridge (2 °C), HPC showed no significant increase, whereas HPC of bottles stored in porch or on a car trunk showed microbial growth [16]. The occurrence of the opportunistic pathogen *P. aeruginosa* in water supplies and bottled water can be a high risk for its consumers [17]. In hospital, *P. aeruginosa* can grow relatively fast in distilled water systems [18]. Soda fountains, which were investigated on microbial contents in a hospital in Germany showed *P. aeruginosa* growth in normal mode without disinfection steps [19]. Contaminated still bottled water caused an outbreak of hospital-acquired *P. aeruginosa* in six intensive care units of a German university hospital [20]. Among other microbial contaminants in bottled water, prevention of *P. aeruginosa* growth is of high interest for consumers as well as for producers of bottled water [21,22]. Worldwide, production sites have similar problems with this type of bacteria within the bottle filling process [[23], [24], [25], [26]]. According to the Codex Alimentarius Austriacus, faecal indicator bacteria such as *E. coli* and other coliform bacteria should not be detectable in 250 mL of bottled water [27]. Pathogen strains like Vero-toxin-producing *E. coli* or enterotoxigenic *E. coli* and the fact that *E. coli* presence indicates faecal contamination of the examined water sample makes an investigation in bottled water still necessary [[28], [29], [30], [31], [32]]. Kerr et al. (1999) investigated the survival of Vero-toxin-producing *E. coli* O157:H7 in natural non-carbonated bottled water. Due to its pathogeny, this strain was considered a serious risk to public health [33]. Bacterial growth of carbonated drinking water produced with in-home carbonation systems showed presence of six coliform strains. Six percent of the collected samples were contaminated with *Enterococcus faecalis* (*E. faecalis*) and 35 % of the inner surface of the bottles was colonized by coliform bacteria [34]. A dependence of bacterial growth to pH-value present in different matrices was shown in recent studies, with *Staphylococcus aureus* (*S. aureus*) being a more sensitive parameter to pH-value than *Staphylococcus epidermidis* [35]. The effect of pH- value on survival of *E. coli* O157 and *E. coli* O121 during desiccation and short-term storage was examined, with *E. coli* O121 being more resistant to these conditions than *E. coli* O157 [36]. For the bacterial contamination with *S. aureus*, pH value dependent growth as well as influence of temperature and surface kind were investigated in studies in the past [[37], [38], [39], [40]]. In the case of carbonated bottled water, the pH-value is predominantly in the acidic range (pH < 7) [41]. An Ethiopian study investigated the mycological and bacteriological quality of bottled water. The carbonated drinking water samples were reported as free of *S. aureus*, *E. coli*, *Salmonella*, *Shigella* and thermotolerant coliforms with no declaration of CO_2_ concentrations [42]. A study reported by Igbeneghu and Lamikanra (2014) led to the conclusion, that small bottling companies often have problems with bacterial contamination [43]. A previous study investigating the behaviour of *S. aureus* and *E. faecalis* in bottled water with different concentrations of CO_2_ showed a reduction of colony forming units over time [44]. The aim of the present investigation is to provide information about the growth of two Gram-negative bacteria (*E*. *coli* and *P. aeruginosa*) in carbonated mineral water “sparkling” (SpMW) and non-carbonated mineral water “still” (StMW) stored in glass bottles over 31 days.

## 2 Material and methods

### 2.1 Preparation of reference strains and artificial contaminated water samples

The reference strains used in this study were obtained from commercial sources: *E. coli* DSM 1103 and *P. aeruginosa* DSM 50071 (Leibnitz-Institute, DSMZ-German Collection of Microorganisms und Cellcultures GmbH, Braunschweig, Germany) and shipped in glass vials. The preparation of the bacterial solution for inoculation of the mineral water bottles was realized with freeze-dried material of the reference strains containing ceramic beads (20 beads per VIABANK® vial) covered in cryopreservative solution (∼10^6^ cfu per bead). Each bead was diluted in 1 L of distilled water. After 10 min 1 mL was transferred into a freshly opened bottle (1 L) and subsequently closed with the screw cap (12 bottles StMW and 12 bottles SpMW). For the determination of the cfu in 1 mL of contaminant solution, three times 1 mL was plated on tryptic soy agar (TSA) (VWR® International GmbH, Vienna, Austria) and counted after 24 h of incubation at 37 °C (*E. coli*, ∼1500 cfu/mL; *P. aeruginosa*, ∼900 cfu/mL).

The values for pH (ÖNORM EN ISO 10523:2012) [45], conductivity (ÖNORM EN 27888:1993) [46] and total organic carbon (TOC) (ÖNORM EN 1484:2019) [47] were measured according to the respective standards with a Memo-Titrator (Metrohm® Inula GmbH, Vienna, Austria), a 712 Conductometer (Methrom® Inula GmbH, Vienna, Austria) and a TOC-Analyzer-TOC Multi N/C (Analytik Jena GmbH, Jena, Germany). The amounts of sodium, potassium, calcium, magnesium, iron and manganese were measured with ICP-OES, iCAP7000 Plus (Thermo Fisher Scientific Inc., Cincinnati, OH, USA) (ÖNORM EN ISO 11885:2009) [48] and values for nitrate, nitrite, chloride and sulphate were determined with a Dionex ICS-1100 Ion Chromatography system (Thermo Fisher Scientific Inc., Cincinnati, OH, USA) (ÖNORM EN ISO 10304-1:2016) [49].

### 2.2 Cultivation

**Fig. 1:**
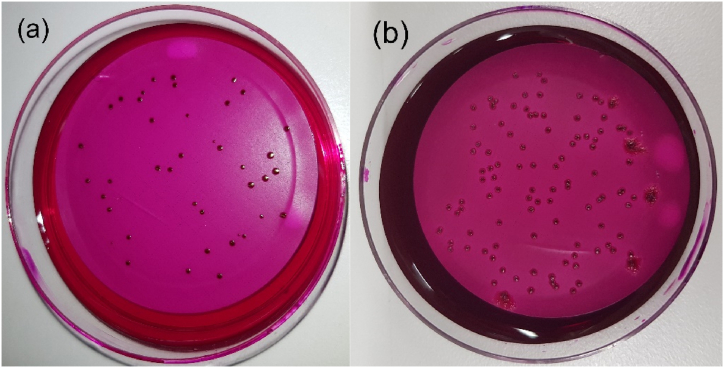
Different appearance of colony-forming units of *E. coli* (a) and *P. aeruginosa* (b), on Endo Agar.

For every filtration and cultivation step in duplicate, a bottle was opened on the respective day. Water samples (10–100 mL) were filtrated through a mixed cellulose ester filter (47 mm diameter, 0.45 μm pore size, EZ-Pak, Merck Chemicals and Life Science GmbH, Vienna, Austria, EZHAWG474) under vacuum, and the filter was subsequently placed on an Endo Agar plate (Merck Chemicals and Life Science GmbH, Vienna, Austria) and incubated for 24 h for *E. coli* and 48 h for *P. aeruginosa* at 37 °C. The counting of CFU's was performed after 24 h for *E. coli* and *P. aeruginosa* with additional counting of cfu's after 48 h. In the case of *P. aeruginosa*, counting after 48 h was impossible due to extensive bacterial growth on the agar plate. For the validation of the respected bacterial colonies, selected cfu's were collected and confirmed by morphology (Fig. 1), cytochrome oxidase testing (BD BBL™ DrySlide Oxidase 231746, BD® Austria GmbH, Schwechat, Austria) and MALDI-TOF VITEK® MS (bioMerieux® Austria GmbH, Vienna, Austria). The determination of the background flora was realized with the filtration of contaminated StMW and SpMW (100 mL) through a mixed cellulose ester filter (47 mm diameter, 0.45 μm pore size, EZ-Pak, Merck Chemicals and Life Science GmbH, Vienna, Austria, EZHAWG474) under vacuum at days seven, 14 and 21 and non-contaminated samples on day one. The filter was subsequently placed on Endo Agar and TSA with a further incubation time of 24 h at 37 °C.

### 2.3 Data analysis

Calculations, standard deviations and charts were prepared using Microsoft Excel.

## 3 Results and discussion

**Fig. 2:**
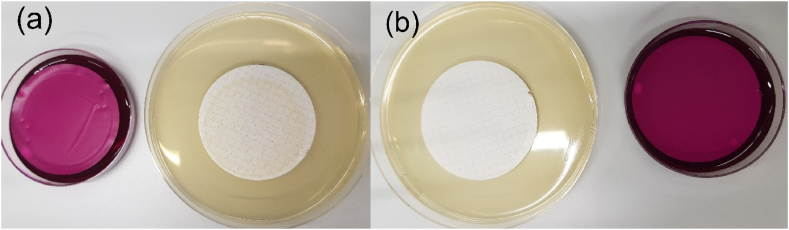
Example of cfu's for background-flora on Endo Agar and Tryptic soy agar of investigated StMW (a) and SpMW (b) of non-contaminated samples on day one after an incubation time of 24 h at 37 °C.

The pH value for StMW was 6.8 ± 0.2 and the electrical conductivity was 410 ± 7 μS/cm. A reduced pH value (5.2 ± 0.1) was observed for SpMW with an electrical conductivity of 417 ± 6 μS/cm. The TOC content of StMW and SpMW was <0.5 mg/L and the value of hydrogen carbonate was 228 mg/L for StMW and 225 mg/L for SpMW. Additional chemical properties of the investigated samples are shown in Table 2 and Table 3. The determination of cfu's for the background flora of non-artificial contaminated water after an incubation for 24 h at 37 °C showed moderate to high values of background-flora for StMW on TSA and no detectable cfu's on Endo agar, which can be seen in Fig. 2a. The samples which were derived from SpMW showed no background-flora after an incubation of 24 h at 37 °C on TSA at all (Fig. 2b). The samples derived at day 14 and 21 artificially contaminated with *E. coli* showed a moderate background flora (day 14, 80 cfu/100 mL; day 21, 69 cfu/100 mL) after the incubation and also one sample derived from a *P. aeruginosa* artificial contaminated bottle on day 21 showed a high background flora (day 21, 280 cfu/100 mL) which was confirmed by MALDI-TOF VITEK® MS measurements. The determination of cfu's regarding background non-target bacteria varied between each bottle. The fact, that different Pseudomonadaceae excluding *P. aeruginosa* were found in the background of the examined water bottles, supports the idea of extensive investigation of bottled water and the filling and capping machinery. In the study of Moreira et al. [50] a slight decrease of *P. aeruginosa* bacterial counts was observed during 20 days in poly vinyl chloride and glass bottles. The authors also indicate, that different types of water, the water containers and the autochthonous flora can affect the cultivation of the target bacteria.

**Table 1:** Mean values for cfu's in StMW and SpMW with *E. coli* and *P. aeruginosa* after an incubation for 24 h at 37 °C. ^*a*^ A volume of 10 mL of sample was filtrated because of high bacterial counts.^b^ A volume of 0.1 mL of the sample was diluted in 9.9 mL of distilled water and was filtrated because of high bacterial counts.

	*E. coli* (∼150 cfu/100 mL)		*P. aeruginosa* (∼90 cfu/100 mL)	
Day	StMW (cfu/100 mL)	SpMW (cfu/100 mL)	StMW (cfu/100 mL)	SpMW (cfu/100 mL)
1	45 ± 1	28 ± 2	86 ± 2	94 ± 4
2	62 ± 4	4 ± 0	170 ± 22	0
7	95 ± 6	0	120 ± 3	0
14	85 ± 2	0	220 ± 6^a^	0
21	28 ± 4	0	380 ± 7^a^	0
31	2 ± 0	0	76000 ± 0^b^	0

**Table 2:** Chemical properties of StMW in mg/L and the hardeness of water in German hardness degree (°dH).

Calcium (Ca^2+)^	Magnesium (Mg^2+^)	Sodium (Na^+^)	Potassium (K^+^)	Iron (Fe)	Manganese (Mn)
54.2	14.1	21.5	1.7	<0.02	<0.05
Ammonium (NH_4_^+^)	Nitrite (NO_2_^−^)	Nitrate (NO_3_^−^)	Chloride (Cl^−^)	Sulphate (SO_2_^−^)	Hardness (°dH)
<0.02	<0.01	10.1	18.6	17.1	10.8

**Table 3:** Chemical properties of SpMW in mg/L and the hardeness of water in German hardness degree (°dH).

Calcium (Ca^2+)^	Magnesium (Mg^2+^)	Sodium (Na^+^)	Potassium (K^+^)	Iron (Fe)	Manganese (Mn)
53.5	14.2	20.5	0.7	<0.02	<0.05
Ammonium (NH_4_^+^)	Nitrite (NO_2_^−^)	Nitrate (NO_3_^−^)	Chloride (Cl^−^)	Sulphate (SO_2_^−^)	Hardness (°dH)
<0.02	<0.01	10.6	21.8	18.2	10.7

**Fig. 3:**
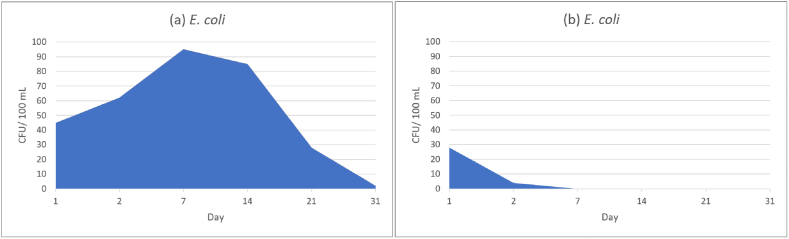
Values of colony-forming units (*E. coli*) over a period of 31 days. StMW (a), SpMW (b).

The values of cfu's after incubation for 24 h at 37 °C are listed in Table 1. During 31 days cfu's for *E. coli* decreased from 45 cfu to 2 cfu with a slight increase after the first day. The increased values of cfu on days two and seven indicate initial stress of the bacteria after preparation and inoculation, which may result in lower numbers (cfu) on the first and second days in a lag-phase manner according to Pletnev et al. (2015) [51]. After day 14 a decrease in cfu's was recognized which supports the findings of a previous published study of Ducluzeau et al.. Ducluzeau et al. described a setting, where 10 mg of faeces were introduced into 500 mL of water with decreasing numbers of *E. coli* over 60 days [52]. In contrast to the high values for TOC after addition of faeces, in the present study, the TOC value was below the detection limit (<0.5 mg/L) which can explain the shorter lifespan of cultivable *E. coli* in the bottles (Fig. 3a) because of a low nutrition environment [53]. In addition, the ability of *E. coli* to form biofilms is low with a better chance to survive in river or pond water than in distilled water samples according to Saima et al. (2021) [54]. With a starting concentration of 150 cfu/100 mL (*E. coli*), SpMW samples showed a fast descrease in cultivable cfu's with only four cfu after the second day and no cultivable cfu's after seven days (Fig. 3b). The decrease of the pH value due to carbonation of the water and cell damaging effects of CO_2_ can explain the findings. In the study of Suehr et al. (2020) it was indicated, that *E. coli* stored at a pH value of five or below showed a high cfu loss per day [36]. Damage may be induced in the bacterial membrane, and/or nucleic acids and may lead to enzyme misfolding or denaturation due to a low pH environment.

The effect of pressurized CO_2_ on bacterial suspensions (*E. coli*) with membrane damage and leakage of intracellular compounds was described in the past. Yao et al. (2014) showed, that after a reaction time of 50 min with 7.5 MPa CO_2_ an inactivation ratio of 100 % was observed for *E. coli* at 25 °C [55]. Although the exact pressure of CO_2_ in SpMW is not known, the results show, that a rapid decrease of cultivable bacteria is observed during 7 days (Fig. 3b).

**Fig. 4:**
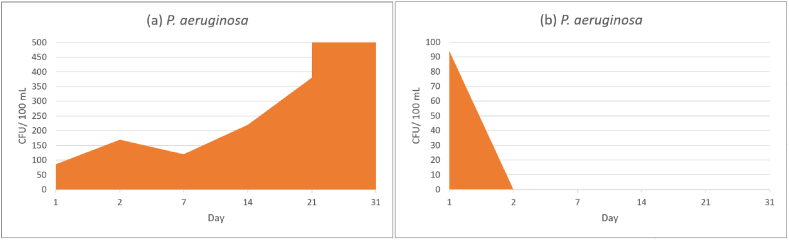
Values of colony-forming units (*P. aeruginosa*) over a period of 31 days. StMW (a), SpMW (b).

In literature it is described that *P. aeruginosa* is a common inhabitant of soil and water [56,57] with an increased occurrence in contaminated areas according to the meta-analysis of Crone et al. (2020) [58]. The *P. aeruginosa* contaminated samples of StMW showed a significant increase in cfu's after the inoculation. *Pseudomonas aeruginosa* showed a better adaptation to the stress during preparation of the solution and inoculation with 86 cfu after the first day of storage. During the period of 31 days, cultivable cfu's of *P. aeruginosa* were increasing to 76 000 cfu on day 31. Dilutions were made (0.1 mL in 9.9 mL of distilled water) to get countable results (Table 1 and Fig. 4a). The low mineralization (410 ± 7 μS/cm) of the examined water samples (StMW) as well as the amounts of sodium, potassium, calcium and magnesium (Table 2) can be compared with classic well and tap water. The physico-chemical properties (Table 2, Table 3) meet the requirements of the Codex Alimentarius Austriacus [32]. The chemical composition of StMW (Table 2) and SpMW (Table 3) shows low amounts of sulphate (SO_2_^−^) and nitrate (NO_3_^−^) while ammonium (NH_4_^+^) and nitrite (NO_2_^−^) Ions were below the detection limit. Nitrate can lead as nitrogen source for bacteria [59] while the genus *Pseudomonas* has been suggested to carry out sulphate reduction under anaerobic conditions [60]. The results for StMW fits to the obtained data for tap water indicated in the study of Moreira et al. [50] where *P. aeruginosa* had a negative mortality rate in tap water bottles. The ability of *P. aeruginosa* to form dense and persistent biofilms on surfaces with extracellular polymeric substances play a critical role in protecting the bacterial community from exogenous stresses and support bacterial growth [61].

The experiment with SpMW showed, that after the first day 94 cfu's were cultivable with a decrease to zero cfu's after the second day of storage. The low pH value (5.2 ± 0.1) and CO_2_ concentration also had a negative effect on cultivable cfu's of *P. aeruginosa* (Fig. 4b).

In their review about the effect of high-pressure CO_2_ on microorganisms’ behaviour, Yu and Chen (2019) collected several studies belonging to this topic [62]. An elevated level of CO_2_ can shift the microbial community structure and lowers the diversity. The microbial metabolism is also affected by CO_2_, which can damage cell structures, leading to cell death.

## 4 Outlook

The influence of different CO_2_ concentrations on the bacterial growth of *E. coli* and *P. aeruginosa* in carbonated water is still unknown. Besides bacterial contaminants, a look on different fungal spores and their ability to survive under an acidic environment with high loads of CO_2_, would provide important information for the beverage industry.

## 5 Conclusion

The problem of contamination during the filling and capping process is still a problem for manufacturers these days. The addition of CO_2_ to bottled water can reduce the culturable bacteria inside the bottles over a certain period of time. The behaviour of *E. coli* in StMW bottles was as expected with a slight decrease of cultivable bacteria over 24 days after an elevation during the first seven days. In contrast to this observation was the behaviour of *P. aeruginosa*, which was detected with increasing numbers to a total count of 76.000 cfu after 31 days in StMW. In SpMW *P. aeruginosa* showed a tremendous decrease in cfu's comparable to the counts of *E. coli*. Although the results of this study indicate a decreasing trend of cfu (*P. aeruginosa* and *E. coli*) in SpMW, more data is necessary to complete the view on the topic of carbonated bottled water. Due to the fact, that a variety of pathogen or facultative pathogen bacteria and fungi can enter the food cycle during manufacturing and packaging, not only CO_2_-addition should be considered for microorganism reduction, but all standards for a proper manufacturing and packaging process should be met besides disinfection procedures.

## Funding

This research did not receive any specific grant from funding agencies in the public, commercial, or non-for-profit sectors.

## Data availability statement

Data will be made available on request.

## CRediT authorship contribution statement

**Michael Schalli:** Conceptualization, Formal analysis, Investigation, Methodology, Software, Supervision, Visualization, Writing – original draft. **Sabine Platzer:** Formal analysis, Investigation, Methodology. **Doris Haas:** Writing – review & editing. **Franz F. Reinthaler:** Supervision.

## Declaration of competing interest

The authors declare that they have no known competing financial interests or personal relationships that could have appeared to influence the work reported in this paper.
